# *In vivo* inhibition of influenza A virus replication by RNA interference targeting the PB2 subunit via intratracheal delivery

**DOI:** 10.1371/journal.pone.0174523

**Published:** 2017-04-05

**Authors:** Daniel Tsung-Ning Huang, Chun-Yi Lu, Pei-Lan Shao, Luan-Yin Chang, Jin-Yuan Wang, Yi-Hsuan Chang, Mei-Ju Lai, Ya-Hui Chi, Li-Min Huang

**Affiliations:** 1 Department of Pediatrics, Mackay Memorial Hospital, Taipei, Taiwan; 2 Graduate Institute of Clinical Medicine, National Taiwan University College of Medicine, Taipei, Taiwan; 3 Department of Pediatrics, National Taiwan University Hospital, National Taiwan University College of Medicine, Taipei, Taiwan; 4 Institute of Biotechnology and Pharmaceutical Research, National Health Research Institutes, Zhunan, Taiwan; University of Hong Kong, HONG KONG

## Abstract

**Background:**

Influenza virus infection is a major threat to human health. Small interfering RNA (siRNA) is a promising approach for the prevention and treatment of viral infections. In this study, we constructed a series of DNA vector-based short hairpin RNAs (shRNAs) that target various genes of the influenza A virus using the polymerase III U6-RNA promoter to prevent influenza virus infection *in vitro* and in a mouse model.

**Results:**

Three sets of DNA vector-based shRNA, two targeting genes encoding the polymerase acidic protein (PA) and one targeting polymerase basic protein 2 (PB2), efficiently inhibited the replication of influenza virus A/WSN/33(H1N1) *in vitro*. We also successfully prevented influenza virus A/WSN/33(H1N1) infection in a C57BL/6 mouse model by intratracheal delivery of anti-PB2 shRNA.

**Conclusions:**

Our findings suggest that the PB2-targeting shRNA plasmid showed potential for use as an RNAi-based therapeutic for influenza virus infection.

## Introduction

Influenza virus infection is one of the most important diseases affecting the respiratory tract [1]. Influenza epidemics affect all age groups and 10~20% of the general population, resulting in up to 48,000 deaths in the United States each year with similar figures in Europe [2, 3]. Currently, influenza vaccines and antiviral drugs are the only available therapeutic strategies to prevent infection. However, vaccines can only offer efficient protection against antigenically similar strains, and they may not be effective against a new potentially pandemic strain. Moreover, the inconvenience of annual vaccinations has led to a low vaccination rate [4]. Furthermore, although several antiviral drugs can reduce the duration of symptoms when given within one or two days of infection, the widespread use of these drugs is limited by concerns about the side effects and possible emergence of drug-resistant variants [5, 6]. Therefore, there is an urgent need for new measures to prevent and treat influenza virus infections.

RNA interference (RNAi) is an emerging technology that specifically inhibits gene expression, and it has been widely used for research in gene function, antiviral and tumor biotherapy [7–9]. Small interfering RNAs (siRNAs), mediators of RNAi, are short (21–25 nt), double-stranded RNA duplexes that inhibit gene expression by inducing sequence-specific degradation of homologous messenger RNA [7]. Several studies have already demonstrated an inhibitory effect on the replication of influenza virus [8–14]. One method to artificially induce siRNA-induced gene silencing is to express short hairpin RNA (shRNA). shRNA is produced from a single transcription unit, and it typically does not activate the interferon response in animal cells [15]. The use of shRNA constructs has further advantages because they are more stable and less expensive than similarly targeted siRNA [16].

The three subunits of RNA polymerase are critical for influenza virus transcription and replication, and they are highly conserved across IAV subtypes. Therefore, the polymerase genes were chosen as the target for RNAi in this study. We constructed a series of DNA vector-based shRNAs under the control of the polymerase III U6-RNA promoter. Three of these shRNAs targeting the polymerase acidic protein (PA) and polymerase basic protein 2 (PB2) genes were found to efficiently inhibit the replication of influenza virus A/WSN/33(H1N1). This effect was demonstrated *in vitro* and *in vivo* using Madin-Darby canine kidney (MDCK) cells, Vero cells, and a C57BL/6 mouse model.

## Results

### Identification of eight target sequences to design the shRNA

We chose eight highly conserved sequences from the PA, PB1, and PB2 genes of influenza virus A/WSN/33(H1N1) and A/Panama/1/68 (H3N2) virus in Influenza Virus Source Databank to design the siRNA (Table 1). Among the eight siRNA sequences, four were chosen to target the PA gene (PAsh1, PAsh2, PAsh3, and PAsh4), two for the PB1 gene (PB1sh1 and PB1sh2), and two for the PB2 gene (PB2sh1 and PB2sh2). These siRNA sequences were further optimized into shRNA and inserted between the U6 promoter and termination sequences (S1 Fig).

**Table 1:** Sequences of siRNA duplexes used in the study.

*Nomenclature*	*Start from*	*Target sequences (from 5’ to 3’)*
**PAsh1**	nt 729–756	GCTACATTGAGGGCAAGCTTTCTCAAAT
**PAsh2**	nt 913–940	GATACCGCTATATGATGCAATCAAATGC
**PAsh3**	nt 1546–1573	GAATGACACCGATGTGGTAAACTTTGTG
**PAsh4**	nt 2062–2089	GAAGCAATTGAGGAGTGCCTGATTAATG
**PB1sh1**	nt 1216–1243	GGAATGATGATGGGCATGTTCAATATGT
**PB1sh2**	nt 2226–2253	GATCATGAAGATCTGTTCCACCATTGAA
**PB2sh1**	nt 140–167	TGAAATGGATGATGGCAATGAAATATCC
**PB2sh2**	nt 1551–1578	GGTCAGTGAAACACAGGGAACAGAGAAA

### shRNA plasmids prevented influenza A/WSN/33 virus infection *in vitro* in MDCK cells

**Fig 1:**
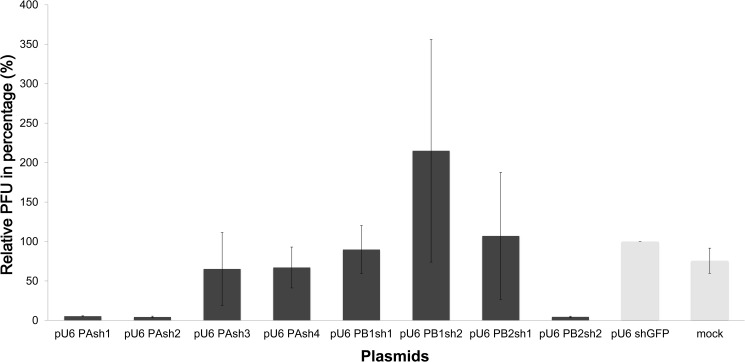
Inhibitory ability of shRNA plasmids against influenza A/WSN/33 virus in MDCK cells. MDCK cells were pretreated with shRNA, which was followed by infection with influenza A/WSN/33 virus at 48 hours. Cell supernatants were harvested at 24 hours post-infection and subjected to a plaque reduction assay to determine the viral titers. The relative reduction was compared to the GFP vector control group. Of the eight investigated shRNAs, only PAsh1, PAsh2, and PB2sh2 plasmids had statistically significant inhibitory effects (*p<0*.*01*).

We tested treatment with 5 μg, 10 μg, and 20 μg of shRNA plasmid in MDCK cells, and an inhibitory effect was only observed at a dose of 20 μg. Of the eight shRNA plasmids investigated in the MDCK cells, only three had the ability to interfere with influenza virus production after testing in triplicate. Compared with the green fluorescent protein (GFP) vector control group, PAsh1, PAsh2, and PB2sh2 significantly inhibited virus replication to an average of 5.3, 4.3, and 4.5%, respectively, (*p<0*.*01*) in plaque assays (Fig 1).

### shRNA plasmids dose-dependently prevented influenza A/WSN/33 virus infection *in vitro* in Vero cells

Although shRNA typically does not activate the interferon response in animal cells [15], it has been reported that gene delivery vectors expressing siRNAs transduced into human tissues may activate immune mechanisms, especially the interferon (IFN) system [17]. To avoid interactions, we tested the PAsh1, PAsh2, and PB2sh2 shRNA treatment in interferon-deficient Vero cells.

**Fig 2:**
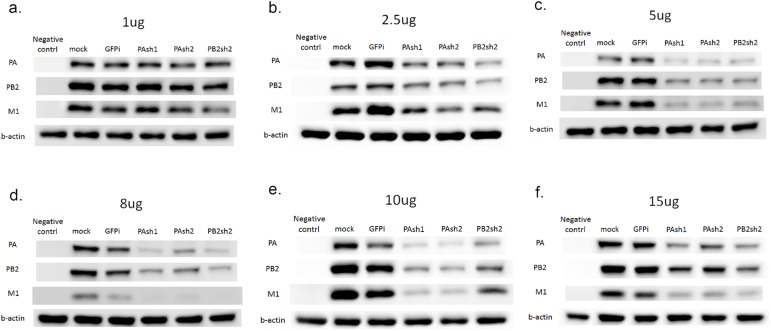
Western blot analysis for shRNAs in Vero cells with influenza A/WSN/33 virus infection. The expression levels of targeted PA, PB2, and untargeted matrix (M1) protein were analyzed by Western blot analysis. We tested treatment with (a) 1 μg, (b) 2.5 μg, (c) 5 μg, (d) 8 μg, (e) 10 μg, and (f) 15 μg of PAsh1, PAsh2, and PB2sh2 shRNA plasmids. The protein expression of the three proteins decreased in a dose-dependent pattern and reached a maximal inhibitory effect at 10 μg.

To determine whether these shRNAs can suppress the expression of targeted PA, PB2 and untargeted matrix (M1) protein, we tested treatment with 1 μg, 2.5 μg, 5 μg, 8 μg, 10 μg, and 15 μg of plasmids. The results (Fig 2) show that the protein expression levels of the three proteins decreased in a dose-dependent pattern and reached a maximal inhibitory effect at 10 μg. With 10 μg as the dose, the PA protein expression level decreased by an average of 51, 57, and 56%, respectively, when cells were co-transfected with PAsh1, PAsh2, and PB2sh2 plasmids. PB2 protein expression decreased by an average of 51, 49, and 37%, and M protein expression also decreased by more than 50% with any shRNA plasmid treatment.

**Fig 3:**
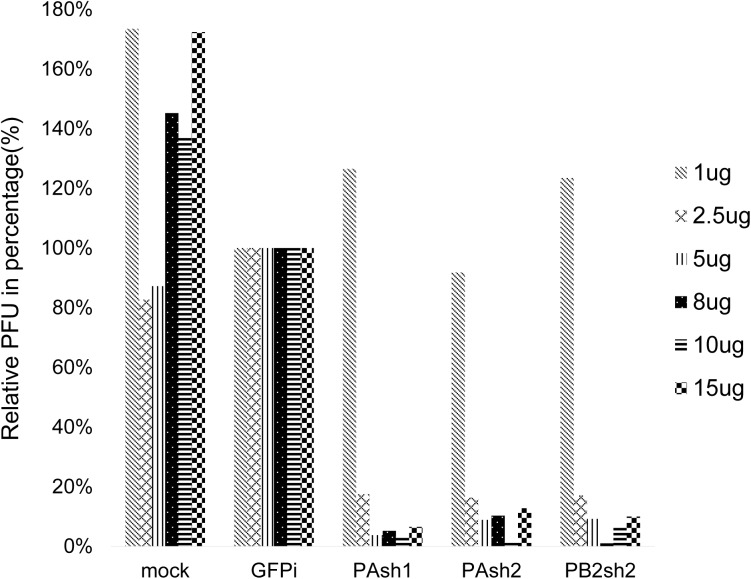
Dose-dependent inhibitory ability of shRNA plasmids against influenza A/WSN/33 virus in Vero cells. Vero cells were pretreated with shRNA, which was followed by infection with influenza A/WSN/33 virus at 48 hours. Cell supernatants were harvested at 24 hours post-infection and subjected to a plaque reduction assay to determine the viral titers. Compared with the green fluorescent protein (GFP) vector control group, PAsh1, PAsh2, and PB2sh2 significantly inhibited virus replication in a dose-dependent pattern and reached a maximal inhibitory effect at 8–10 μg, which depended on the shRNA that was used.

The inhibitory ability of the three shRNA plasmids, illustrated by plaque-forming units (PFUs), is shown in Fig 3. Compared with the green fluorescent protein (GFP) vector control group, PAsh1, PAsh2, and PB2sh2 significantly inhibited virus replication. A gradient of an increasing inhibitory effect was observed as the dosage of shRNA increased, and it reached a maximal inhibitory effect at 8–10 μg, which depended on the shRNA that was used (Fig 3).

### shRNA plasmids had no inhibitory effect on mutant protein expression levels in the rescue experiment

We further conducted a rescue experiment to elucidate the possible phenomenon of off-target silencing. We constructed four pcDNA3 plasmids, two containing wild-type (WT) and two mutant (mut) viral proteins, and individually co-transfected them with inhibitory shRNAs. The silent mutations were made in the targeted regions of PAsh1, PAsh2, and PB2sh2 shRNA, as shown in the Methods section.

**Fig 4:**
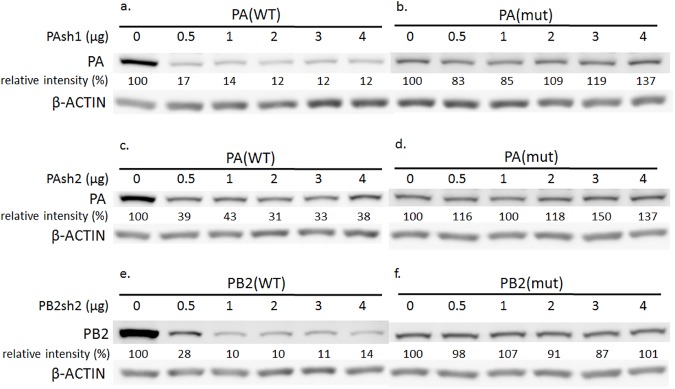
Inhibitory ability of shRNA treatment in wild type versus mutant PA and PB2 proteins. Four plasmids, two containing wild-type (WT) and two mutant (mut) viral proteins, individually co-transfected with inhibitory shRNAs. (a) PAsh1-shRNA and PA (WT) plasmids, (b) PAsh1-shRNA and PA (mut) plasmids, (c) PAsh2-shRNA and PA (WT) plasmids, (d) PAsh2-shRNA and PA (mut) plasmids, (e) PB2sh2-shRNA and PB (WT) plasmids, and (f) PB2sh2-shRNA and PB (mut) plasmids. The shRNA inhibitory effects on wild type (WT) PA and PB2 proteins were evident in Western blots, while no inhibitory effects were seen in the mutant (mut) protein expression levels. The knockdown effects of the shRNAs on wild type proteins were concordant with the administered dose.

The shRNA inhibitory effects on wild type (WT) PA and PB2 proteins were evident in Western blots, while no inhibitory effects were seen in the mutant (mut) protein expression levels (Fig 4). The knockdown effects of the shRNAs to wild type proteins were concordant with the administered dose. This rescue experiment further ruled out the possibility of off-target effects of the shRNAs in our study.

#### shRNA plasmids did not knockdown clinical seasonal influenza H1N1 and H3N2 virus infections *in vitro* in Vero cells

**Fig 5:**
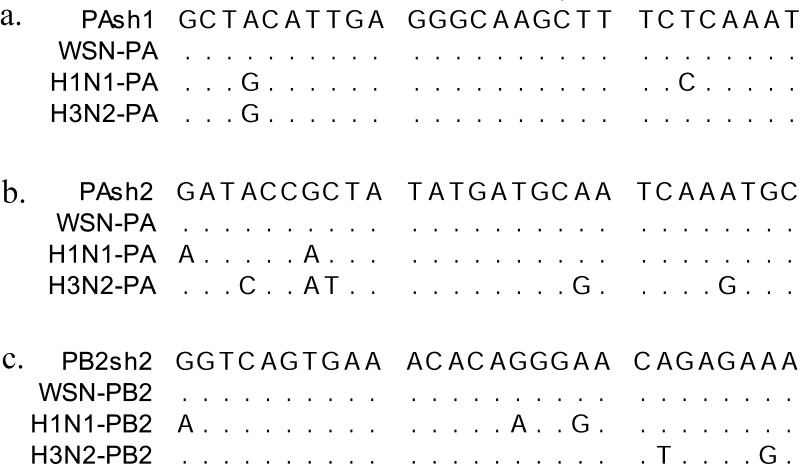
Alignment of the shRNAs sequences with two clinical trains (seasonal H1N1 and H3N2). We employed two clinical strains of influenza virus (seasonal H1N1 and H3N2) and aligned the targeted sequences with the shRNAs. The shRNA sequences do not perfectly match the two clinical strains.

**Fig 6:**
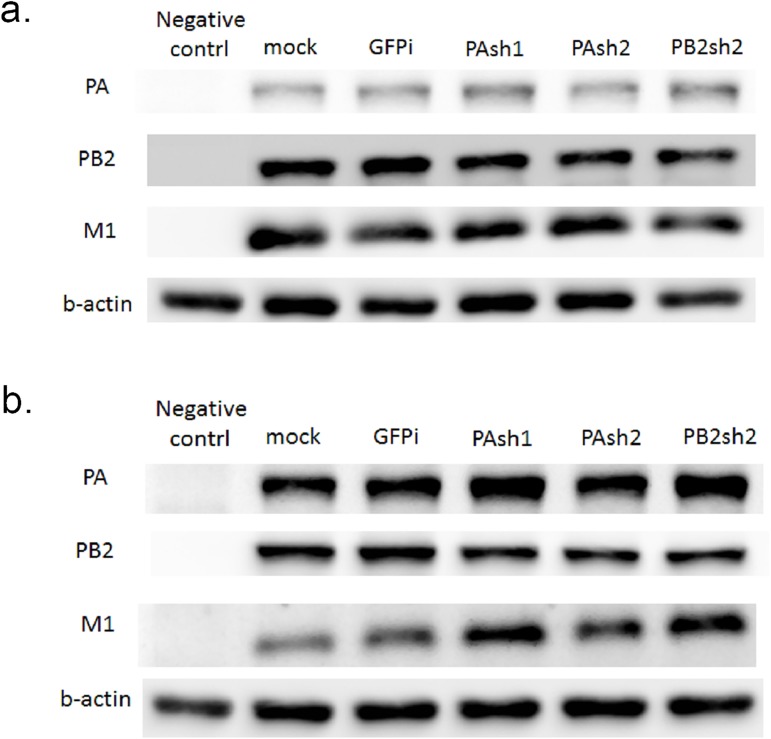
Inhibitory ability of shRNA treatment against seasonal H1N1 and H3N2 influenza virus in Vero cells. We tested treatment with 8μg of shRNA plasmid in Vero cells and co-transfected the cells with two clinical influenza isolates (seasonal H1N1 and H3N2). The results showed no obvious inhibitory effects on PA, PB2, or M protein expression in Western blot analyses.

We employed two clinical strains of influenza virus (seasonal H1N1 and H3N2) in the study and aligned the targeted sequences with the three designed shRNAs (i.e., PAsh1, PAsh2 and PB2sh2, Fig 5). The shRNA sequences do not perfectly match the two clinical H1N1 and H3N2 strains. We tested the cross-strain effect of the shRNAs with the two clinical H1N1 and H3N2 strains in Vero cells. No significant inhibitory effect on PA, PB2, or M protein expression was observed by Western blot analysis (Fig 6).

### Design and localization of the intratracheal shRNA delivery model in mice showed promising results

**Fig 7:**
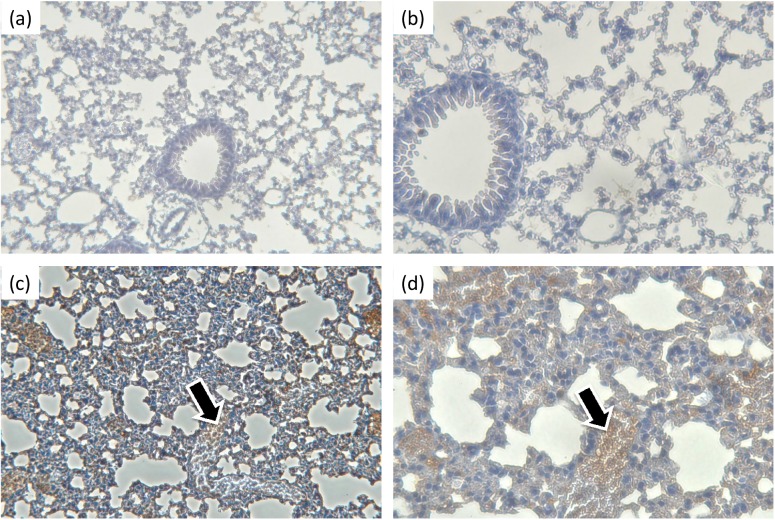
Biodistribution of plasmids in the lungs by intratracheal delivery. The intratracheal model was performed by administering the plasmids through a 2.5-mm human otoscope specula that was placed directly into the trachea via the mouth. Forty-eight hours after plasmid delivery, GFP proteins were observed in lung sections by immunohistochemistry (IHC) staining (black arrows). Negative controls at (a) 200X and (b) 400X with IHC staining and (c) 200X and (d) 400X.

GFP-expressing plasmids in polyethylenimine (jetPEI) were delivered intratracheally into 8-week-old female C57BL/6 mice. The GFP protein was translated in the lung and could be visualized by light microscopy 48 hours later with immunohistochemistry (IHC) staining (Fig 7). This indicated that our plasmids were successfully delivered to and translated in the lungs.

To test the virus inhibitory effect of shRNA *in vivo*, mice were pretreated with PAsh1 (n = 4), PAsh2 (n = 4), PB2sh2 (n = 4), and control vector (n = 4) 24 hours before inoculation with influenza virus A/WSN/33(H1N1), and the mice were sacrificed 72 h post-infection. Influenza virus titers were assessed by RT-PCR targeting the matrix gene using the WHO real-time RT-PCR protocol. In all treatment groups, the average virus titers were reduced in the lungs compared to the control group (Table 2) (p < 0.05).

**Table 2:** Mean influenza A/WSN/33 virus titers in murine lungs treated with shRNAs.

*Treatment*	*Mean virus titer (per 100 mg lung)*	*p-value*
**GFPi**	1.37E+08 ± 2.15E+07	—
**PAsh1**	9.66E+07 ± 1.14E+07	0.01
**PAsh2**	9.35E+07 ± 1.36E+07	0.01
**PB2sh2**	6.77E+07 ± 6.53E+06	0.003

### One shRNA plasmid prevented influenza virus A/WSN/33 (H1N1) infection *in vivo*

**Fig 8:**
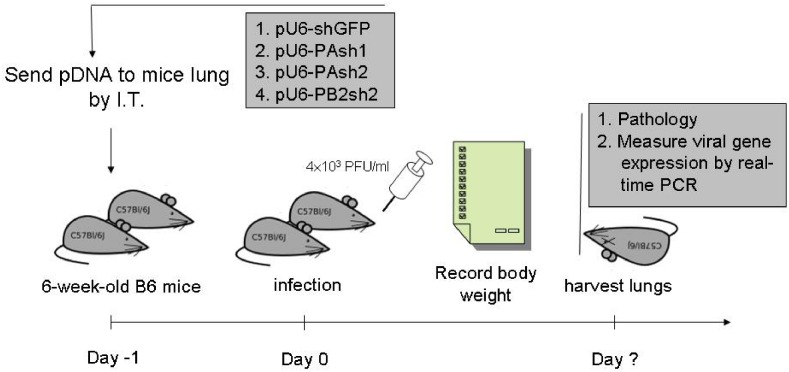
Timeline for studying the shRNA intratracheal delivery model in mice. shRNA plasmids were mixed with jetPEI with a N/P ratio of 5. Twenty micrograms of plasmids in 50 μL of carrier were administered intratracheally through a 2.5-mm human otoscope specula placed directly into the trachea. The mice were inoculated with 4×10^3^ PFU/mL influenza virus 24 hours after shRNA transfection. The body weights of the mice were recorded daily, and the lungs were harvested at the time of death or upon euthanasia at day 14. I.T.: intratracheal.

**Fig 9:**
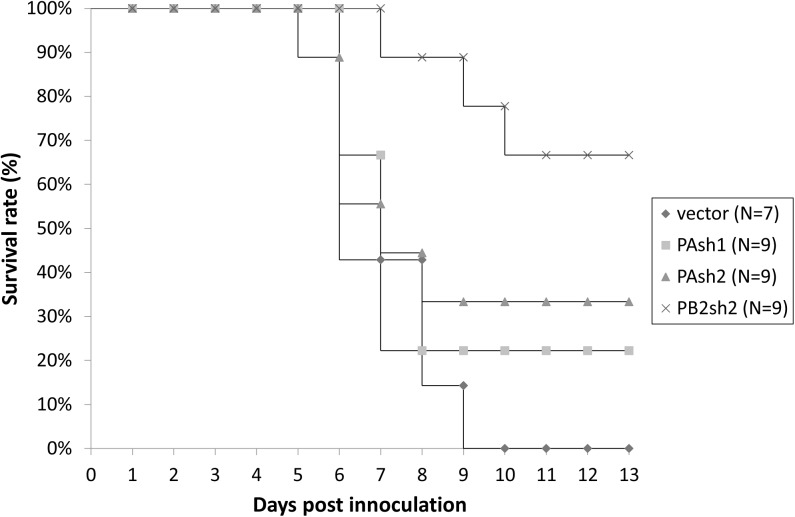
Survival rates of the mice treated prophylactically with shRNA plasmids by intratracheal delivery. Thirty-four mice were treated prophylactically with PAsh1, PAsh2, or PB2sh2 shRNA plasmids via intratracheal administration. The mice were pretreated 24 hours before inoculation with influenza virus. On day 14, only the PB2sh2 shRNA plasmid demonstrated a statistically significant protective effect (*p = 0*.*008*).

The mice were pretreated with PAsh1 (n = 9), PAsh2 (n = 9), PB2sh2 (n = 9), and control vector (n = 7) 24 hours before inoculation with influenza virus A/WSN/33(H1N1) (Fig 8). On day 14, six of the nine (67%) mice in the PB2sh2-treated group (*p = 0*.*008*) survived, while only two and three survived in the PAsh1- and PAsh2-treated groups, respectively. The mice in the control group all succumbed to infection within 10 days (Fig 9).

### shRNA plasmids could not treat ongoing influenza virus infection

To determine whether the shRNAs were therapeutically effective, the mice were infected with influenza virus and simultaneously transfected with shRNA plasmids. However, all mice died within 10 days of infection (data not shown), suggesting that shRNA plasmids may be therapeutically ineffective.

## Discussion

The genome of influenza A virus consists of eight ribonucleoprotein complexes (vRNPs) that occur as ribonucleoprotein (RNP) particles with four core viral polypeptides, which are the PB1, PB2, and PA subunits of an RNA-dependent RNA polymerase and a single-strand RNA binding nucleoprotein (NP). It is believed that the PB2 protein plays an important role in nuclear localization and genome replication via interactions with other influenza proteins [18].

In this study, we found that three different shRNA sequences targeting the PA and PB2 genes inhibited influenza A/WSN/33 virus replication *in vitro*. We also found that pretreatment with one PB2-targeting shRNA could effectively reduce the mortality rate of the mice after virus infection. To the best of our knowledge, this is the first study to report successful PB2-targeting shRNA-based anti-influenza therapy in a mouse model.

There are several possible reasons why only one in three shRNAs worked *in vitro* but failed *in vivo*. For example, in regard to *in vivo* delivery of shRNA, the condition is much more complicated than in cell culture, considering the stability, aggregation with mucosal proteins, uptake by phagocytes, and diffusion in the extracellular matrix. To improve the *in vivo* efficiency for siRNA-based therapy, conjugating or complexed siRNAs with peptides, lipids, polymers, and polycations may be helpful in other studies [19]. A cocktail of multiple siRNAs can also be effective against other viral strains and subtypes [20].

siRNA therapy has been considered a promising approach for anti-influenza therapy since Ge *et al* [5] and Tompkins *et al* [14] reported remarkable experimental evidence *in vitro* and in mice. Both studies used shRNA targeting the NP, PA, or PB1 genes and treated the experimental animals intravenously or intranasally. Ge *et al* also showed that shRNA, expressed by a lentivirus DNA vector, could prevent influenza A/Puerto Rico/8/34-induced pneumonia in mice. A number of subsequent studies have shown that siRNA targeting different RNA segments can inhibit the replication of highly pathogenic avian influenza virus H5N1 [10, 12, 13] and various influenza A (H1N1) viruses *in vitro* and *in vivo* [9, 11, 21–23].

Several *in vivo* siRNA-based anti-influenza studies suggested that the siRNAs are only effective when inoculated prior to viral challenge. For example, Ge *et al* showed that the viral load was reduced ≈100-fold at 24 hours after 3,000 pfu PR8 virus infection in mice that were intravenously pretreated with NP-shRNA compared with luc shRNA. In another mouse study by Tompkins *et al*, increased survival rates were observed in mice that were intravenously pretreated with NP- or PA- siRNAs 16–24 hours before PR8 viral challenge (5 × 102 TCID50, estimated 350 pfu). Zhou *et al* injected M2+NP siRNAs into mice, prophylactically, 18 hours before a large dose of H1N1 viral challenge (10^6^ TCID50, estimated 7 x 10^5^ pfu). Li *et al* intravenously injected PB1-shRNAs into the mice eight hours before PR8 viral challenge, and Rajput *et al* injected NS1-siRNAs to the mice 24 hours ahead of challenging them with 2 x 10^6^ pfu PR8 virus. Compared with previous *in vivo* studies, the amount of 4×10^3^ PFU/mL influenza A/WSN/33 virus that we used was comparable to, or even lower than, the reported lethal dose for mice. The negative result in our post-challenge experiments may be due to the insufficient level of shRNAs produced in the lung (the production rate peaks 48–72 hours post transfection), which cannot combat the short replication cycle of influenza virus (approximately 6 hours [24]).

In conclusion, our findings suggest that the PB2-targeting shRNA plasmid showed potential for use as an RNAi-based therapeutic for influenza virus infection.

## Materials and methods

### Ethics statement

The procedures and use of the animals were approved (protocol number: 20100379) by the Institutional Animal Care and Use Committee (IACUC) of National Taiwan University Hospital (Taipei, Taiwan). The temperature of the environment was controlled between 20–25°C with 60–70% humidity. The ventilation rate was set to 10–15 times per hour. Five female mice shared a cage with a toy house inside. We fed the mice with LabDiet5001 standard diet (LabDiet®, St. Louis, MO). Extra gentle handling was used when no procedure was executed.

After virus inoculations, the mice were visited every day, and their body weights, appearances, appetites, activities, and abnormal behaviors were recorded. When the mice were observed to have >10% body weight loss, analgesic intramuscular meperidine 2.0 mg/100 g BW was given optionally according to IACUC guidelines. The mice were euthanized with CO_2_ via inhalation when there was more than 20% body weight loss or when they did not eat or drink for more than 24 hours. For the invasive treatments, including intratracheal delivery of the shRNAs, we used Zoletil (Virbac, Carros CEDEX, France) 250 μg + xylazine 125 μg intramuscularly for anesthesia.

### Cell culture

Madin-Darby canine kidney (MDCK) cells (ATCC CCL-34) and Vero cells (ATCC CCL-81) were maintained in Eagle’s Minimum Essential Medium (MEM) and 293T cells (ATCC CRL-1573) in Dulbecco’s Modified Eagle’s Medium (DMEM), which were supplemented with 10% fetal calf serum, 100 U/mL penicillin, 100 μg /mL streptomycin and 0.25 μg/mL amphotericin B.

### Plasmid construction

The pSilencer 2.1-U6 puro plasmid vector (Applied Biosystems/Ambion, Austin, TX, USA) was used to express specific shRNAs. To increase the efficiency of transfection, we modified the vector by removing sequences from Sfi I (3318) to *Xba* I (4193), reducing the vector size from 4455 bp to 3598 bp (S1 Fig).

Eight highly conserved sequences from PA, PB1, and PB2 genes (PAsh1, PAsh2, PAsh3, PAsh4, PB1sh1, PB1sh2, PB2sh1, and PB2sh2) were selected for and inserted between the U6 promoter and termination sequences. The sequences were conserved for influenza A/WSN/33 (H1N1) virus and A/Panama/1/68 (H3N2) virus in the Influenza Virus Source databank (https://www.ncbi.nlm.nih.gov/genome/viruses/variation/flu), and they were screened by the Ambion web-based criteria. We utilized a website for efficient antiviral siRNA design (sivirus.rnai.jp) to design our siRNAs. Eight oligonucleotides were designed in sense-loop-antisense sequences into which the loop sequence *Xho I* recognition site (ACTCGAGA) was inserted (S1 Fig). The siRNA target sequences for the influenza A/WSN/33 virus are listed in Table 1. These oligonucleotides were annealed and ligated to the BamHI and HindIII sites of the modified pSilencer 2.1-U6 puro siRNA expression vector to obtain the expression plasmids.

Small RNA (≤200 nt) was isolated using a *mir*Vana^TM^ miRNA isolation kit (Ambion). Mature double-stranded siRNAs (PAsh1 and -sh4, PB1sh1 and -sh2, and PB2sh1 and -sh2) were quantified by a two-step real-time RT-PCR following the protocol provided with the TaqMan^®^ MicroRNA Assays (Ambion).

### Transfection and virus infection *in vitro*

Influenza A virus, A/WSN/33 (H1N1), was obtained from the Taiwan Centers for Disease Control. Clinical specimens (H1N1 and H3N2) were collected from influenza-infected hospitalized patients at Taiwan National University Hospital, Taipei, Taiwan. Cell culture harvests were stored at -70°C until used in the reverse transcription-polymerase chain reaction (RT-PCR) assay.

The virus was grown on MDCK/Vero cells infected at a multiplicity of infection of 0.01. Then, 8×10^4^ to 2×10^5^ MDCK/Vero cells were seeded in 6-well plates. Twenty-four hours later, the cells were transfected with eight different shRNA expression plasmids.

We tested treatment with 5 μg, 10 μg, and 20 μg of shRNA plasmids in MDCK cells, and inhibitory effects were only observed at a dose of 20 μg. Each 20 μg of shRNA plasmid was mixed with jetPEI (Polyplus-Transfection Inc., France), a water-soluble linear polyethylenimine derivative, at a nitrogen:phosphorus weight ratio (N/P ratio) of 5 at room temperature for 20 minutes. The transfected MDCK cells were infected 48 hours later with influenza A/WSN/33 virus, which was followed by plaque reduction assays for 24 hours to measure the virus titers. A control plasmid expressing GFP was similarly introduced into MDCK cells, which was followed by virus infection.

The same method was used in Vero cells with a different polymer for transfection. We tested treatment with 1 μg, 2.5 μg, 5 μg, 8 μg, 10 μg, and 15 μg of shRNA plasmids in Vero cells. Each shRNA plasmid was mixed with TransIT^®^-LT1 Transfection Reagent (Mirus Bio LLC, WI) and Opti^®^-MEM (Life Technologies CO., Ltd., Taiwan) at a nitrogen:phosphorus weight ratio (N/P ratio) of 3 at room temperature for 30 minutes.

### Plaque reduction assay

A plaque reduction assay was performed to determine the infectivity of the virus. A total of 2×10^5^ MDCK/Vero cells were seeded in 12-well plates. After 24 hours, cell monolayers were infected with virus-containing supernatant harvested from MDCK/Vero cells that had been previously infected with the A/WSN/33 virus in the presence or absence of shRNA. A 10-fold serial dilution of virus preparation was added to each well and incubated in a CO_2_ incubator at 37°C. One hour post-infection, the supernatant was removed, and 1 mL of overlay medium containing 5% agarose gel in MEM supplemented with 2% fetal bovine serum (agarose:medium ratio 1:9) was added to each well. Forty-eight hours later, the cell monolayers were fixed in 4% formalin for 2 hours. Viral plaques were stained with 0.03% methylene blue for 2 minutes, and the number of plaques was counted under light microscopy. Infectivity titers were interpreted as the plaque forming unit (PFU)/mL.

### Rescue experiment

To elucidate the phenomenon of off-target effect, we constructed four pcDNA3 plasmids, two containing wild-type (WT) and two mutant (mut) viral proteins, and individually co-transfected with shRNA containing plasmids. The silent mutations were made in the targeted regions of PAsh1, PAsh2, and PB2sh2 shRNA as described below.

We used the NEBaseChanger tool to choose mutant sites and the Q5®Site-Directed Mutagenesis Kit (New England Biolabs Inc.) to make the mutations. The mutant sites on the relevant targeting sequences of PAsh1, PAsh2, and PB2sh2 are listed below.

1. PA (WT)-GCTACATTGAGGGCAA***GC***T***T***TCTCAAAT (719~946 = 28 bp); GATACCG***C***T***A***TATGATGCAATCAAATGC (903~930 = 28 bp); PA (mut)-GCTACATTGAGGGCAA**AT**T**A**TCTCAAAT (735, 736, 738 bp); and GATACCG**T**T**G**TATGATGCAATCAAATGC (910, 912 bp). The two mutations were made in the same plasmid.
2. PB2(WT)-GGTCAGTGA***A***AC***A***CAGGGAACAGAGAAA (1551~1578 = 28 bp) and PB2(mut)-GGTCAGTGA**G**AC**T**CAGGGAACAGAGAAA (1560, 1563 bp).

A total of 1x10^6^ 293T cells were seeded in 6-well plates and incubated overnight. The cells were then co-transfected with PAsh1-shRNA and PA (WT) plasmids, PAsh1-shRNA and PA (mut) plasmids, PAsh2-shRNA and PA (WT) plasmids, PAsh2-shRNA and PA (mut) plasmids, PB2sh2-shRNA and PB (WT) plasmids, and PB2sh2-shRNA and PB (mut) plasmids. Different wells had the same 0.5 μg level of protein expressing plasmids, but they were added in increasing levels of shRNA plasmids. We used empty vector (pSilencer 2.1-U6 puro) to balance the total plasmid DNA to 4.5 μg/well.

We tested treatment with 0 μg, 0.5 μg, 1 μg, 2 μg, 3 μg, and 4 μg of shRNA in 293T cells. Each shRNA plasmid was mixed with TransIT®-LT1 Transfection Reagent at a nitrogen: phosphorus weight ratio (N/P ratio) of 3. Twenty-four hours after co-transfection of plasmids, we performed cell lysis to extract protein. Western blot analysis was used to identify each protein. We then compared the relative intensity of each band between wild type and mutant sequences.

### Localization of the plasmids in the lung by immunohistochemical staining

GFP plasmids were mixed with jetPEI and intratracheally delivered into 8-week-old female C57BL/6 mice. The lungs were harvested from each mouse 48 hours later; then, they were fixed with 3% paraformaldehyde solution for 16 to 18 hours and embedded in wax. Samples were cut into 4-μm slices for immunohistochemical staining with mouse anti-GFP antibody (Millipore MAB3580) (Millipore, Billerica, MA, USA) and the N-Histofine® MOUSESTAIN KIT (Nichirei, Tokyo, Japan). The distribution of the GFP protein was observed under light microscopy and photographed (Zeiss, Germany) (Fig 5).

### shRNA transfection in mice using an intratracheal delivery model

shRNA plasmids were mixed with jetPEI as described above. Each 20 μg of plasmid in 40 μL of jetPEI (N/P ratio = 5) was intratracheally delivered into 8-week-old female C57BL/6 mice (National Taiwan University Laboratory Animal Center, Taipei, Taiwan). The intratracheal model was performed by administering the plasmid through a 2.5-mm human otoscope specula (Welch Allyn, USA) placed directly into the trachea from the mouth (Fig 6) to ensure controlled delivery of precise plasmid volumes.

### Virus infection in mice

Twenty-four hours after shRNA transfection (pSilencer 2.1-U6 puro plasmid), the mice were intratracheally inoculated with 4×10^3^ PFU/mL influenza A/WSN/33 virus in 40 μL of PBS. In our *in vivo* study, mice were given only one dose of shRNA, and no plasmid DNA was given after virus challenge. The body weight of the mice was recorded daily. Lungs from the mice were harvested at the time of death or upon euthanasia at day 14. The cumulative survival rate was calculated at day 14. Left side lungs were sent for pathological examination of the inflammatory cells, and right side lungs were sent for the measurement of influenza viral titers.

To determine the influenza virus titers, 100 mg of the lungs were homogenized with 1.5 mL of TRIzol. RNA was extracted using RNeasy (Qiagen) according to the manufacturer’s instructions. Influenza virus titers were assessed by RT-PCR targeting the matrix gene using the WHO real-time RT-PCR protocol (Inf A Forward. GAC CRA TCC TGT CAC CTC TGA C. Inf A Reverse. AGG GCA TTY TGG ACA AAK CGT CTA) [25].

### Statistical analysis

Statistical analysis was performed using SAS version 9.3 (SAS Institute, Cary, NC). In the study of *in vivo* shRNA plasmid prevention in the mice (Fig 7), multiple comparisons of each shRNA were analyzed using log rank tests adjusted for multiple comparisons by Dunnett’s test. A *p* value < 0.05 was considered statistically significant.

## Supporting information

S1 Fig Plasmid construction and secondary structure of the pU6-PB2sh2 shRNA. pU6-PB2sh2 is shown as an example of the construct design and shRNA secondary structure. Complete targeting sites for PB2sh2 corresponding to nucleotides 1551 to 1578 of influenza A virus (A/WSN/1933 (H1N1)) segment 1, the PB2 gene for RNA polymerase complex. [GenBank: CY034139.1]
(TIF)
