# Supplementary figures and images for: In vivo inhibition of influenza A virus replication by RNA interference targeting the PB2 subunit via intratracheal delivery
Source: PLoS One. 2017 Apr 5;12(4):e0174523. doi: 10.1371/journal.pone.0174523 (PMC5381882; doi:10.1371/journal.pone.0174523)

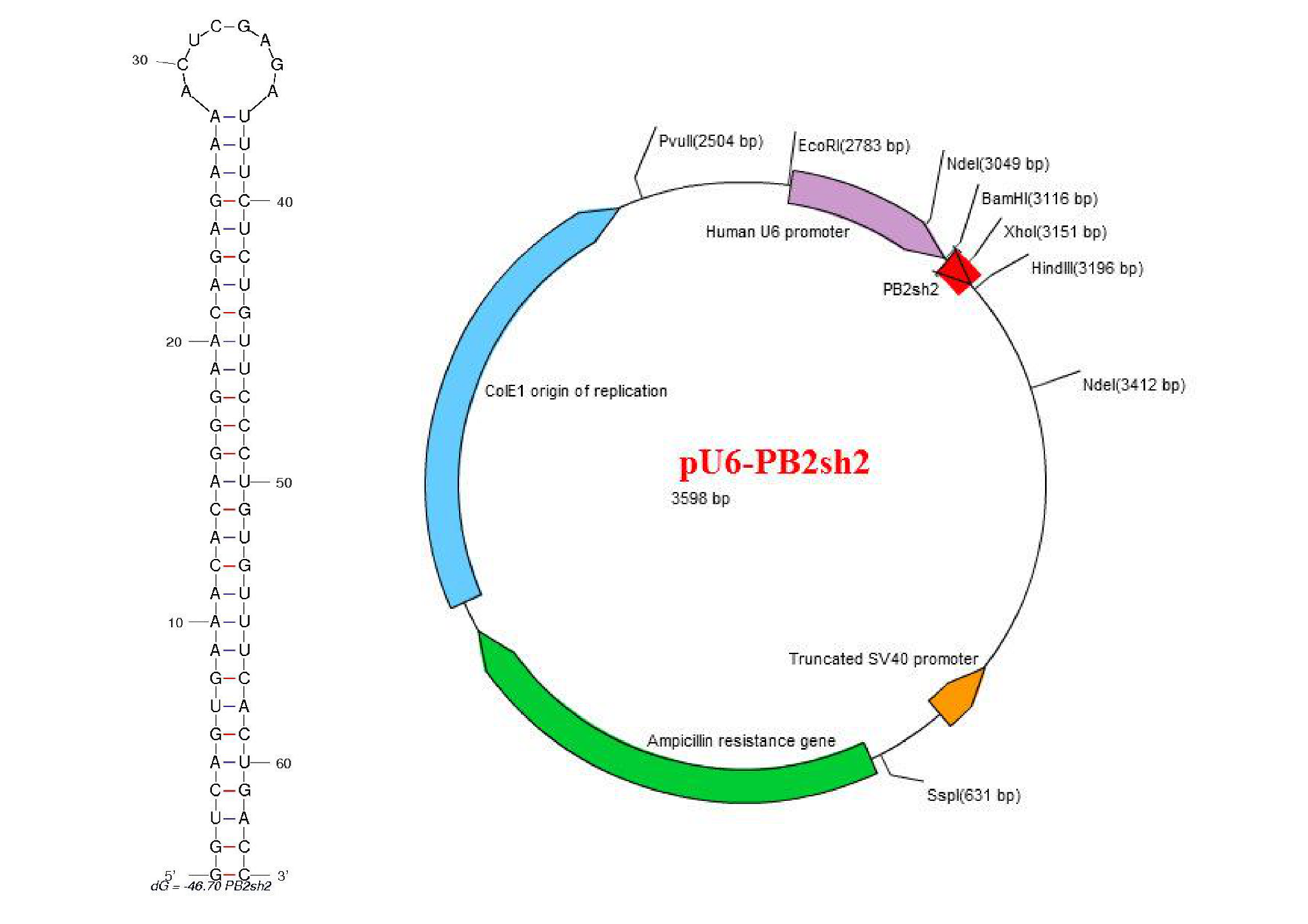

Supplement: S1 Fig — pU6-PB2sh2 is shown as an example of the construct design and shRNA secondary structure. Complete targeting sites for PB2sh2 corresponding to nucleotides 1551 to 1578 of influenza A virus (A/WSN/1933 (H1N1)) segment 1, the PB2 gene for RNA polymerase complex. [GenBank: CY034139.1] (TIF) [file pone.0174523.s001.tif]
